# The Sources of Hookworm Infection and the Manner in Which Infection Takes Place*Read by invitation at the annual meeting of the Mississippi State Medical Society, May 16, 1910.

**Published:** 1910-10

**Authors:** C. C. Bass

**Affiliations:** New Orleans, La.


For Texas Medical Journal.

### The Sources of Hookworm Infection and the Manner In Which Infection Takes Place.*

BY C. C. BASS, M. D., NEW ORLEANS, LA.

Certain biologic facts relative to uncinariasis must be known, if we would understand well the sources of infection. Different species of uncinaria have been found parasitic in various lower animals; namely, dogs, sheep, cattle, horses, pigs, cats, foxes, seals, etc. These are distinct from those infecting man and have not been found in man.

Two species infect man—anchylostoma duodenalis or the Old World hookworms and uncinaria americana or the New World hookworms. The latter were first identified by Stiles in material from Porto Rico, but were later found widespread throughout the Southern United States, and are practically the only species found in this country infecting man except a few imported cases of anchylostomiasis.

Both of these varieties have been shown experimentally to be capable of entering the skin of lower animals, but they have not reached adult life in the intestinal canal of any animal besides man except possibly the chimpanzee, the gibbon and the gorilla. They reach adult life or the reproductive stage only in the intestinal canal of man where they lay eggs which pass out with the feces.

The eggs do not hatch in the intestinal canal for lack of oxygen and possibly for other reasons. A man can not, therefore, directly infect himself. Moreover, it has been shown by Looss working with the Old World hookworm, Claude A. Smith working with the American variety, and by others, that the young worms can not infect until they have reached a certain stage (the encysted stage) of their life, which requires at least four or five days after they hatch, which itself requires twenty-four to thirty-six hours.

Every case of hookworm infection must come from another infected individual, and, inasmuch as the eggs are contained in his feces only, the real source of every hookworm infection must be the excrement of such infected persons.

The number of eggs passed with each stool of a heavily infected patient is enormous. Leichtenstern estimated the number passed

---

*Read by invitation at the annual meeting of the Mississippi State Medical Society, May 16, 1910.

in one of his cases to be over 4,000,000. This was infection with Old World hookworms. I counted the eggs in one decigram of feces from a severe case of uncinaria americana infection, originating in Louisiana, and calculated the number in the entire stool to be 4,490,000

It is the habit of many of our rural population to stool on the ground in any convenient place. Every day he plants thousands and even millions of eggs capable of developing into larvæ of the most infectious parasites, ready to attack anybody who comes in contact with them.

Rains dilute the feces and mix them with dirt, putting them in the most favorable condition for hatching. Uncinaria eggs will not hatch in liquid feces nor in undiluted feces except near the surface where the material has partially dried out. They will not hatch in perfectly dry feces. If feces containing eggs are diluted with dirt, sand, or other material and favorable conditions of moisture, warmth (temperature between 27° and 45° C.) and shade exist, the microscopic embryos begin to hatch out and crawl about in the diluted feces in twenty-four hours. In two to three days they shed their skin somewhat like snakes do, and certain developmental changes occur in their anatomy. This is the first ecdysis.

After about five days from the time hatched out the organism begins a second ecdysis or stage of development, but this time it remains inside of the skin, which it has cast or retracted from and is spoken of as "encysted." Up to this stage the animal takes food, but it takes no more while encysted. This stage ends the life of the worm outside of the body. In this encysted condition these microscopic larvæ may live three months or longer under favorable conditions of moisture, shade and temperature; and are now able to infect man but were not before. They resist wonderfully well unfavorable conditions and agents, such as chemicals, but are killed by freezing. They require nothing but moisture to keep them alive. They live well in water or in moist earth, in which they come near the surface or go deeper with the rise and fall of moisture. They are killed by the hot sun, and require shade to live in.

Drinking water may become a source of infection, but the probabilities of getting any considerable number of larvæ in this way seems pretty remote on account of the amount of contamination that would be required, and especially in view of the fact that the larvæ soon sink in water. Water containing larvæ would have to be stirred up just before it is drunk to become a source of infection.

Leichtenstern showed that infection by mouth can occur by administering per os capsules containing the larvæ. In about six weeks ova of the parasites appeared in the feces of the patient. These experiments with anchylostoma have been repeated and confirmed with uncinaria americana by Claude A. Smith and others in this country. Food that has been contaminated with infected mud or water or that has been handled with dirty infected hands may become a source of infection.

Smith noted the habit the encysted larvæ have of crawling up along the sides and cover of a culture dish, which suggests another way in which certain food may become infected. Larvæ may crawl upon the leaves, stalk or fruit of such vegetables as lettuce, celery, strawberries, etc., that are eaten raw and may be swallowed. No ordinary washing could be expected to remove all of them. The frequent practice of defecating in the barnyard where the feces would be mixed with the barnyard manure and frequently used to fertilize vegetable and other crops, may possibly be a source of infection.

Looss, in 1898, while experimenting with cultures of anchylostoma larvæ accidentally found that getting the culture on his hands produced a dermatitis. Later, on finding ova in his feces, he concluded that he had been infected through the skin. In 1901 he proved the correctness of his theory by experiments. His work was not confirmed by Grassi, Pieri and Noe, only one of whom developed the disease after all had dropped fluid containing larvæ on their skin. They concluded that this case and Looss' were instances of accidental infection by mouth. Looss then repeated his experiments and established beyond all doubt that the larvæ enter the skin and finally reach the intestines, where they develop to adult life. He placed seven drops of heavily-infected water on the arm of a man whose feces showed that he was not then infected. The typical dermatitis shortly developed. The next day the arm was swollen. In a week the swelling subsided. In seventy-one days ova were found in his feces. He shaved the back of a dog and placed on it some mud heavily infected with uncinaria larvæ. The animal was properly bandaged to prevent mouth infection and after two hours the mud was removed and the area thoroughly scrubbed with absolute alcohol. The mud contained only one-sixth as many larvæ as it did when applied to the dog's back. The dog died in ten days, and at autopsy an enormous number of larvæ were found on and in the walls of the small intestines.

Looss infected two other puppies, one by feeding infected milk and the other by the skin. They both died in a few days and their small intestines contained enormous numbers of young larvæ.

In 1902, Bentley found in a water sore, called in this country ground itch or toe itch, a young worm which he thought to be anchylostoma duodenale. He then made the following experiments: A culture of sterilized soil plus feces containing eggs was made and another one containing no eggs; both were properly incubated at ordinary room temperature. A part of each was gently dried for eight hours. Six hours drying had previously been found sufficient to kill the larvæ. After remoistening the specimens that had been dried, samples of the four specimens were applied to the wrists of the subjects of experiment for eight hours, and then they were removed. Within fifteen hours after the application, erythema and a papular eruption appeared over the spot where the living larvæ had been applied; within twenty-four hours an itching vesicular eruption had developed, followed by pustules exactly resembling those found in the lesions of ground itch. In all the other cases no such lesions appeared. A re-examination of the mud showed that the live worms had disappeared but that the dead ones were still present. The live worms had, therefore, apparently entered the skin and their entry had been followed by lesions and symptoms similar to those of water sore or ground itch.

These experiments with anchylostoma have been paralleled by the brilliant work of Claude A. Smith, and the results found to hold good for uncinaria americana. He placed mud containing encysted larvæ on the arm of a man not previously infected and allowed it to remain one hour. In eight minutes itching was complained of, and on removing the soil a macular eruption was present. The next day the wrist was swollen. On the following day a vesicular eruption was present and much itching was complained of. On the fifth day the vesicles had become confluent, the swelling increased and the axillary glands enlarged and tender. The twelfth day no signs of the dermatitis remained. On the eighth day an attack of sore throat with fever developed. During the next three weeks an uneasy feeling about the stomach was complained of. Ova appeared in the stools at the middle of the seventh week.

A small particle of mud was applied, without his knowledge, to the prepuce of a man about to be circumcised. In four minutes he remarked that he felt as though a fly was crawling over the part and in nine minutes longer said he felt as though his prepuce was being pricked by needles. In four minutes the larvæ

had left the mud, as shown by subsequent examination, but under a low-power lens the skin was seen covered with them.

He, also, was able to produce lesions resembling those of ground itch by applying an alcoholic extract of uncinaria larvæ, though aqeous and ethereal extracts gave negative results.

Sections of the skin removed and hardened even in a few minutes after infectious larvæ had been applied show numerous larvæ down in the hair follicles, sweat pores, and already actually in the tissues. In sections prepared after a longer time has elapsed, many larvæ can be found deep in the skin and subcutaneous tissue, some of which may now be found in capillary blood-vessels. They possess wonderful ability to plow through any kind of tissue, in the encysted stage, but will not attempt to penetrate the skin before this stage is reached. Once in the blood stream they are carried to the right heart. Sections have been made in which the larvæ were found in blood in the heart cavity. From the heart they are carried by the blood stream to the lungs. Here they are caught in the capillaries, because they are so much larger than the smallest capillaries of the lungs and can not pass through. Meeting with obstruction, they again exercise their ability to penetrate tissue and soon get into the bronchial tubes.

Ashford and King infected a young guinea pig with a very heavily-infected culture, and in three hours the animal died. Autopsy showed one lung solidified with blood and the other one contained many larger or smaller hemorrhagic spots. Sections showed numerous uncinaria larvæ in these accumulations of blood.

After the larvæ reach the bronchi they are carried to the mouth either as a result of the normal constant outward current of the bronchial and tracheal mucous membrane or by coughing. No doubt, many of them are now spit out but some are swallowed. More or less larvæ would be swallowed according to whether an individual spits out what he coughs up or whether he expectorates little. Habits vary much in this regard, as is well known. No doubt this is an important factor in determining the extent of infection in different individuals equally exposed, and, in view of this idea, it seems wise to advise patients who have ground itch not to swallow their sputum.

Larvæ that are swallowed pass through the stomach, resisting the gastric juice just as they resist other chemicals, sulphuric acid, bichloride of mercury, etc., and when they reach the small intestine, the natural home of the parasite, animal instinct or something else induces them to penetrate the mucous membrane of the gut.

Four or five days after the larvæ reach the small intestine another ecdysis begins, whereby they acquire a buccal capsule and another skin is cast. With this capsule the worm fastens on to the mucous membrane by sucking in a plug of epithelium, which furnishes his nourishment.

In another four or five days the last ecdysis begins and the last skin is shedded. The worms about one-fifth inch long now grow rapidly, and in six to eight weeks from the original infection they begin to lay a few eggs, which are discharged with the feces of the patient.

That infection through the skin is possible from short contact with infected mud and water is proven. Every step and stage of this process has been demonstrated by several independent workers.

Accepting this fact, it is only necessary to consider whether, in the life of the class of people who have hookworm disease, their skin is more exposed to dirt and water containing human feces, or whether they are more likely to eat and drink food and water containing appreciable amounts of this material to see the greater opportunity for skin than mouth infection.

Those who have lived in the country districts have only to call to mind the barefoot children running around the yard, barnyard and fields, walking and playing in mud and water and sand, often more or less polluted with human feces, to realize that almost daily they are exposed to such infection.

Adults frequently go barefoot during the summer months, thus exposing themselves. Leaky shoes may also allow infection to get to the skin.

Whether the infectious larvæ may not frequently go through a wet shoe is worth considering. Bearing on this question, I put some charcoal and feces mixed on a thin piece of leather from the arm part of a lady's kid glove that had previously been soaked in water. A place was selected not containing a seam and apparently free from holes. Some mud made of sterilized dirt and water was put on the opposite side. Three hours afterward the mud was carefully removed and examined. Four living larvæ and two dead ones were found during a long search.

If larvæ pass through leather experimentally, it is probable that they do practically in many instances in which people carry infected mud on their shoes a whole day at a time. They would cause recognizable skin lesions only when large numbers were present. A man might get a few worms in this way every wet spell, and, as they live for years, the accumulation year after year might amount to considerable infection. As I see it now, this is, to me, sufficient explanation for the cases of hookworm disease we see who have not gone barefoot and who have not had ground itch. They usually occur only where ground itch and hookworm disease are common.

SUMMARY.

The real source of every case of hookworm disease is eggs of the parasite in the feces of infected people.

Young worms hatch from these and after four or five days become encysted and infectious.

These can gain entrance to the body through the mouth, on dirty hands, in food and water, or through the skin in any way in which the infected material comes in contact with it, chiefly as a result of going barefoot; possibly also infection through wet shoes.

The fact that one or many attacks of ground itch is a part of the life history of almost every boy living in an infected locality, whereas those from non-infected localities do not know what the disease is; that experimental hookworm infection through the skin produces lesions identically like ground itch, and the further fact that in infected localities the skin is almost daily exposed to soil and water polluted with human excreta, whereas drinking water and food are far less exposed, all indicate that skin infection is the practical and almost the only one that actually occurs.

The worms penetrate the skin chiefly through the hair follicles and sweat pores, enter the blood stream, pass through the right heart to the lungs, into the bronchi, thence to the mouth and now are swallowed, pass through the stomach to the small intestine, where they attach themselves to the mucous membrane and develop to adult life.

---

Dr. Brumby has resigned as State Health Officer and President of the Board of Health, and becomes Medical Director of the Equitable Life Insurance Company, of San Antonio (not of Houston, as stated last month). Dr. Fred Combes, late of Brownsville, is president of the company. Dr. Brumby has made a zealous Health Officer. Our best wishes go with him. It is said that Mr. Campbell will leave the filling of the vacancy to his successor, Colquitt, who becomes Governor in January.

---

Dr. Jno. B. Elliott, Jr., Tulane, has been appointed to the Chair of Medicine, made vacant by the resignation of Professor Dock, who has gone to Washington University Medical College, St. Louis.

